# Increased EBNA1-specific antibody response in primary-progressive multiple sclerosis

**DOI:** 10.1007/s00415-024-12763-w

**Published:** 2024-12-12

**Authors:** Manuel Comabella, Harald Hegen, Luisa M. Villar, Konrad Rejdak, Augusto Sao-Avilés, Malina Behrens, Jaume Sastre-Garriga, Neus Mongay, Klaus Berek, Sergio Martínez-Yelamos, Francisco Pérez-Miralles, Ahmed Abdelhak, Franziska Bachhuber, Hayrettin Tumani, Jan Lycke, Pere Carbonell-Mirabent, Adrián Valls-Carbó, Igal Rosenstein, Roberto Alvarez-Lafuente, Tamara Castillo-Triviño, David Otaegui, Sara Llufriu, Yolanda Blanco, Antonio J. Sánchez-López, Antonio García-Merino, Nicolás Fissolo, Lucía Gutiérrez, Javier Villacieros-Álvarez, Enric Monreal, Heinz Wiendl, Xavier Montalban, Jan D. Lünemann

**Affiliations:** 1 Servei de Neurologia, Centre d’Esclerosi Múltiple de Catalunya (Cemcat), Institut de Recerca Vall d’Hebron (VHIR), Hospital Universitari Vall d’Hebron, Universitat Autònoma de Barcelona, Barcelona, Spain; 2 Center for Networked Biomedical Research on Neurodegenerative Diseases (CIBERNED)-ISCIII, Madrid, Spain; 3 Department of Neurology, Medical University of Innsbruck, Innsbruck, Austria; 4 Departments of Neurology and Immunology, Hospital Universitario Ramón y Cajal, Instituto Ramón y Cajal de Investigacion Sanitaria, Madrid, Spain; 5 Department of Neurology, Medical University of Lublin, Lublin, Poland; 6 Department of Neurology with Institute of Translational Neurology, University Hospital Münster, Münster, Germany; 7 Department of Neurology, Bellvitge University Hospital, Barcelona, Spain; 8 Neuroimmunology Unit, València University and Polytechnic Hospital La Fe, València, Spain; 9 Department of Neurology, Ulm University, Ulm, Germany; 10 Division of Neuroinflammation and Glial Biology, Department of Neurology, University of California San Francisco, San Francisco, USA; 11 Fundación INCE (Iniciativa Para Las Neurociencias), Madrid, Spain; 12 Department of Clinical Neuroscience, Institute of Neuroscience and Physiology at Sahlgrenska Academy, University of Gothenburg, Gothenburg, Sweden; 13 Environmental Factors in Degenerative Diseases Research Group, Hospital Clínico San Carlos, Instituto de Investigación Sanitaria del Hospital Clínico San Carlos (IdISSC), Madrid, Spain; 14 Neurology Department, Hospital Universitario Donostia, San Sebastián, Spain; 15 Multiple Sclerosis Unit, Biodonostia Health Research Institute, San Sebastián, Spain; 16 Neuroimmunology and Multiple Sclerosis Unit, Service of Neurology, Hospital Clinic and Institut d’Investigacions Biomèdiques August Pi i Sunyer (IDIBAPS), University of Barcelona, Barcelona, Spain; 17 Neuroimmunology Unit, Puerta de Hierro-Segovia de Arana Health Research Institute, Madrid, Spain; 18 Biobank, Puerta de Hierro-Segovia de Arana Health Research Institute, Madrid, Spain; 19 Department of Neurology, Hospital Universitario Ramón y Cajal, REEM, IRYCIS, Universidad de Alcalá, Madrid, Spain

**Keywords:** Multiple sclerosis, Primary progressive, Virus, Epstein–Barr virus, Human cytomegalovirus

## Abstract

**Background and objectives:**

The impact of viral infections on disease susceptibility and progression has predominantly been studied in patients with relapse-onset MS (RMS). Here, we determined immune responses to ubiquitous viruses in patients with primary progressive MS (PPMS).

**Methods:**

Antibody responses to Epstein–Barr virus (EBV), specifically to the latent EBV nuclear antigen 1 and the lytic viral capsid antigen VCA, human herpesvirus 6 (HHV-6), human cytomegalovirus (HCMV), and measles virus were determined in a cohort of 68 PPMS patients with a mean follow-up of 8 years and compared with 66 healthy controls matched for sex and age.

**Results:**

Compared with controls, PPMS patients showed increased humoral immune responses to the EBV-encoded nuclear antigen-1 (EBNA1), but not to the lytic EBV capsid antigen (VCA) or to other viral antigens. Seroprevalence rates for HCMV were significantly higher in PPMS. Antiviral immune responses at baseline did not correlate with disability progression over time.

**Discussion:**

Elevated immune responses toward EBNA1 are selectively increased in people with primary progressive disease, indicating a link between EBNA1-targeting immune responses and the development of both RMS and PPMS. Our data also suggest that chronic HCMV infection is associated with progressive MS.

**Supplementary Information:**

The online version contains supplementary material available at 10.1007/s00415-024-12763-w.

## Introduction

Genetic and environmental factors determine susceptibility to the development of multiple sclerosis (MS) and contribute to the progression of the disease [1]. Epidemiological studies provided strong evidence for consistent environmental risk-associations such as the increased susceptibility for MS following Epstein–Barr virus (EBV) infection and a protective role for human cytomegalovirus (HCMV) [2, 3]. These associations have primarily been studied in patients with relapse-onset MS (RMS), as this is the most prevalent manifestation and course of the disease. Only about 15% of the patients develop a progressive disease course from onset, termed primary progressive multiple sclerosis (PPMS). Given major epidemiological and clinical differences between RMS and PPMS in terms of sex predominance, age at onset, initial clinical presentation, rate of disability progression and response to immunotherapy, it has long been debated whether PPMS is a distinct disease entity or whether it just represents part of the heterogeneous clinical disease spectrum [4]. Epidemiological and clinical differences between RMS and PPMS have also led to the question whether the disease courses have distinctive risk factors. Monozygous twins can be concordant or discordant for disease courses and no clear genetic differences have been found between RMS and PPMS, indicating that the disease course is not predominantly genetically determined [5].

## Methods

In the present study, we determined immune responses to ubiquitous viruses, as potential environmental trigger of the disease, in a large cohort of 68 patients with PPMS recruited from 12 European MS centers, compared to demographically matched healthy control donors (HD) (Suppl. Table 1). All patients fulfilled the 2017 revisions of the McDonald criteria [6]. Median (interquartile range—IQR) follow-up time for patients from baseline was 8.0 (7.0–10.7) years. Only one patient (1.5%) was treated during follow-up. EDSS scores were recorded at baseline (i.e. sampling time point), 2 and 6 years, and at the time of last visit. Short-term disability progression was defined as an increase of at least 1 point in the EDSS if baseline EDSS ≤ 5.0 and 0.5 points if baseline EDSS ≥ 5.5 during the first 2 years. Taking into account that most of the patients would fulfill this progression criterion at medium and long term, to assess disability progression at these time points, progression rates were computed by dividing EDSS changes by the time on follow-up between baseline and 6 years for medium-term disability progression and between baseline and the time of the last visit for long-term disability progression. Then, medium- and long-term progressors were defined as those patients displaying progression rates above the 75th percentile of disability progression. Virus antigen-specific IgG responses were assessed in sera using commercially available ELISA kits according to the manufacturers’ recommendations. The following kits were used: EBNA1 (# RE58741, Tecan IBL International GmbH, Hamburg, Germany), EBV-CA (#El 2791-9601G, Euroimmun, Lübeck, Germany), CMV (#El 2570-9601G, Euroimmun, Lübeck, Germany), HHV6 (#KA1457, Abnova, Taoyuan City, Taiwan), Anti-Measles Virus (#El 2610-9601G, Euroimmun, Lübeck, Germany). All samples were frozen upon venipuncture, not previously thawed and analyzed together at one time point. Seroprevalence rates were compared between PPMS and HD by a chi-square test, antibody-responses were analyzed using the non-parametric Mann–Whitney *U* test. Univariable logistic regressions were performed to assess the association between antiviral immune responses at baseline and disability progression at short term (2 years), medium term (4 years), and long term (at the time of last follow-up). Given the exploratory nature of our study, we did not apply correction algorithms for multiple testing. Anonymized data will be shared upon reasonable request. The study was approved by the corresponding Hospital Ethics Committee according to the ethical standards laid down in the 1964 Declaration of Helsinki, and participants gave written informed consent. Anonymized data will be shared upon reasonable request.

## Results

Seroprevalence rates for HCMV were significantly higher in PPMS patients compared to HDs matched for sex and age (76.5% for PPMS vs. 50% for HD; *p* = 0.001) (Table 1). Although not statistically significant, trends towards higher seroprevalence rates for the EBV-encoded antigens EBNA1 and VCA were observed in PPMS patients. Seroprevalence rates against HHV-6 and measles virus were similar between both groups.

**Table 1:** Seroprevalence rates against viruses in patients with PPMS and healthy donors

Viruses	PPMS	Healthy donors	*p*-value
EBV-VCA	68 (100%)	63 (95.5%)	0.075
EBV-EBNA1	67 (98.5%)	61 (92.4%)	0.088
HCMV	52 (76.5%)	33 (50.0%)	**0.001**
HHV-6	38 (55.9%)	42 (63.6%)	0.360
Measles	66 (97.1%)	61 (92.4%)	0.228

*EBV* Epstein–Barr virus, *EBNA1* Epstein–Barr nuclear antigen 1, *HCMV* human cytomegalovirus, *HHV-6* human herpesvirus 6, *VCA* viral capsid antigen

Significant *p*-values are shown in bold

**Fig. 1:**
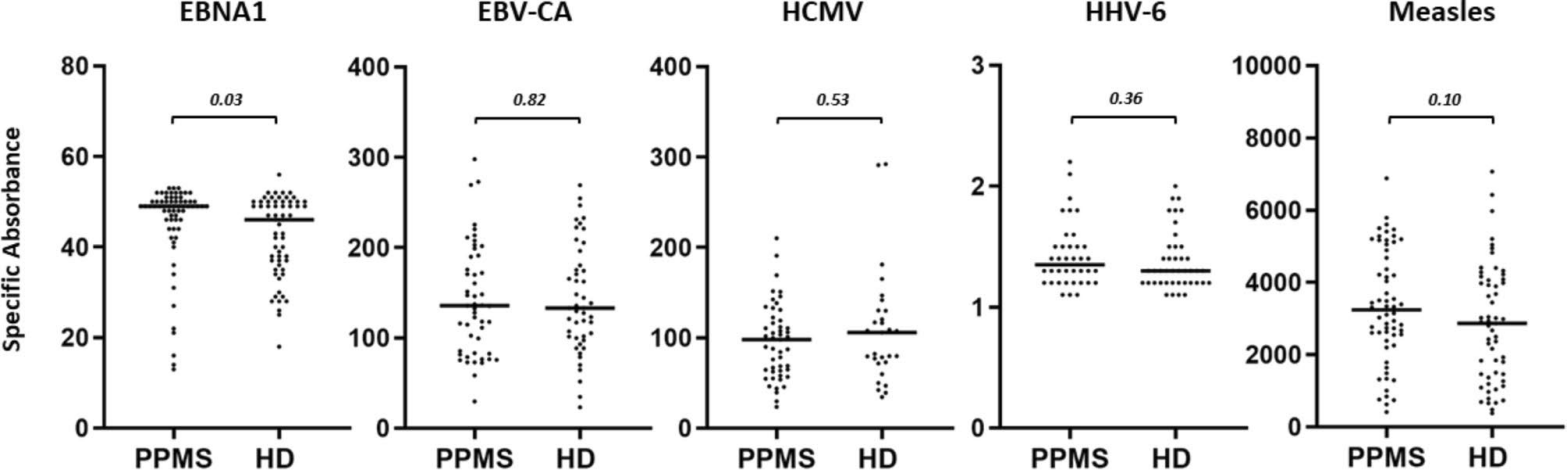
Selective increase of EBNA1-specific IgG response in PPMS patients graphs show the distribution of IgG immune responses against ubiquitous viruses in patients with PPMS compared to healthy donors (HD) matched by sex and age. The non-parametric Mann–Whitney *U* test was used to compare antibody responses between the 2 groups. *EBV* Epstein–Barr virus, *EBNA1* Epstein–Barr nuclear antigen 1, *HCMV* human cytomegalovirus, *HHV-6* human herpesvirus 6, *VCA* viral capsid antigen

Seropositive PPMS patients showed a selective and significant increase of IgG responses towards EBNA1 compared to healthy EBV carriers (Fig. 1). Antibody responses to the lytic EBV-encoded capsid antigen as well as to proteins derived from other viruses were similar in patients and controls (Fig. 1). Taking advantage of the long follow-up of the PPMS cohort, we next investigated whether antiviral immune responses at baseline were associated with disability accrual over time. As shown in Table 2, antiviral antibody responses at baseline were not associated with disability progression at short term (2 years), medium term (6 years), or long term (at last follow-up).

**Table 2:** Association between antiviral immune responses and disability progression at short, medium, and long term in patients with PPMS

Viruses	Short term	Medium term	Long term
EBV-VCA	HR = 0.997 (0.991–1.003); *p* = 0.350	HR = 0.999 (0.992–1.006); *p* = 0.802	HR = 1.001 (0.995–1.007); *p* = 0.729
EBV-EBNA1	HR = 1.072 (0.981–1.172); *p* = 0.124	HR = 1.015 (0.945–1.089); *p* = 0.687	HR = 1.032 (0.959–1.112); *p* = 0.401
HCMV	HR = 1.002 (0.993–1.011); *p* = 0.671	HR = 1.008 (0.998–1.018); *p* = 0.131	HR = 1.000 (0.990–1.011); *p* = 0.946
HHV-6	HR = 2.043 (0.154–27.022); *p* = 0.588	HR = 2.830 (0.179–44.770); *p* = 0.460	HR = 0.623 (0.045–8.580); *p* = 0.723
Measles	HR = 1.000 (1.000–1.000); *p* = 0.570	HR = 1.000 (1.000–1.000); *p* = 0.592	HR = 1.000 (1.000–1.000); *p* = 0.749

Data are expressed as hazard ratios (HR) and 95% confidence intervals after univariable logistic regression analysis

*EBV* Epstein–Barr virus, *EBNA1* Epstein–Barr nuclear antigen 1, *HCMV* human cytomegalovirus, *HHV-6* human herpesvirus 6, *VCA* viral capsid antigen

## Discussion

The selective increase of EBNA1-specific antibody responses in PPMS patients without a concomitant increase of immune responses to other EBV-VCA and to proteins derived from other ubiquitous viruses is consistent with previous studies performed in patients with clinically isolated syndromes (CIS), in patients with RMS and in healthy individuals who will develop MS [7–10]. Suggesting specificity for MS**,** EBNA1-targeting antibody responses are reported to be unchanged in patients with myelin oligodendrocyte glycoprotein-antibody associated disease (MOGAD) [11] and a broader anti-EBV T cell receptor repertoire has recently been described to be specifically associated with MS but absent in aquaporin 4-antibody positive neuromyelitis optica spectrum disorder (NMOSD), MOGAD, or in Susac’s syndrome [12].

Only a few studies have investigated EBV- and antiviral immune responses as a potential risk factor for MS by disease course. Farrell et al. reported higher EBNA-1 but lower EBV-VCA IgG titers in RMS (*n* = 25) versus PPMS (*n* = 25) patients, HD sera or immune responses to viruses other than EBV were not investigated [13]. Ingram et al. found that EBNA1-specifc IgG responses are not significantly increased in neither PPMS (*n* = 25) nor active (*n* = 25) and stable (*n* = 25) RMS compared to HD (*n* = 25) but appreciated that the study was likely underpowered to detect significant differences [14]. In a population-based case–control study, comprising 7520 RMS cases, 540 PPMS cases and 11,386 HDs matched by age, sex and residential area, Hedström et al. [13] reported that EBNA1-specific IgG responses are increased in both patients RMS and PPMS compared to HD. Immune responses to viral antigens other than EBNA1 were not investigated in the latter two studies and none of aforementioned investigations included longitudinal data on disability progression.

Our data show that EBNA1-specific immune responses are increased not only in RMS but also in PPMS. The increase appears to be predominantly associated with EBNA1 as antibody responses to other viral antigens, including the lytic EBV capsid antigen, were similar in PPMS and HD. As reported for RMS [15], increased immune responses to EBNA1 at baseline were not correlated with disability progression in PPMS. These data support the notion that EBNA1-specific immune responses potentially contribute to the development of both RMS and PPMS, but do not predict disease progression after onset. This does not exclude the possibility that changes in antiviral immune responses over time may reflect clinical disease activity, severity or progression in PPMS.

The finding of higher seroprevalence rates of HCMV in PPMS compared to demographically matched HD appears surprising since HCMV seropositivity and increased antibody responses to HCMV are associated with protection from the development and progression of RMS [2, 3]. Aging is a biological factor strongly associated with both HCMV seropositivity and progressive MS. Chronic HCMV infection is believed to contribute significantly to immunosenescence, an age-related loss of innate and adaptive immune system proficiencies, thought to accelerate MS progression [16]. Our study, therefore, provides incentive to conduct larger studies on the potential role of EBV and HCMV infection as risk factor specifically related to primary progressive MS.

## Supplementary Information

Below is the link to the electronic supplementary material.

Supplementary file1 (DOCX 16 kb)
