# Supplementary material for: Increased EBNA1-specific antibody response in primary-progressive multiple sclerosis
Source: J Neurol. 2024 Dec 12;272(1):26. doi: 10.1007/s00415-024-12763-w (PMC11638268; doi:10.1007/s00415-024-12763-w)
Supplement: Supplementary file 1 — Supplementary file1 (DOCX 16 kb) [file 415_2024_12763_MOESM1_ESM.docx]

**Supplementary Table 1.** Demographic and clinical characteristics of PPMS patients and healthy controls.

| Characteristics | PPMS | Control donors |
| --- | --- | --- |
| N | 68 | 66 |
| Age (years)^a,b^ | 52.2 (9.1) | 52.5 (9.4) |
| Male / female (% men) | 26 / 42 (38.0) | 27 / 39 (40.9) |
| Disease duration (years)^c^ | 3.9 (2.0 - 8.3) | - |
| Follow-up time (years)^d^ | 8.0 (7.0 - 10.7) | - |
| EDSS at baseline | 3.5 (2.5 - 5.5) | - |
| EDSS at 2 years^e^ | 4.0 (3.0 - 6.5) | - |
| EDSS at 6 years^f^ | 6.0 (5.0 - 7.0) | - |
| EDSS at last visit | 6.3 (4.8 - 7.1) | - |
| Treatment during follow-up (n (%))^g^ | 1 (1.5) | - |

#### Data are expressed as median (interquartile range) unless otherwise stated. ^a^Data are expressed as mean (standard deviation). ^b^Refers to age at sample collection. ^c^Refers to the time between disease onset and sample collection. ^d^Refers to the time between samples collection and the time of last visit. ^e,f^Information missing in 6 (8.8%) and 10 (14.7%) patients, respectively. ^g^One patient was treated with interferon-beta for 2 years after lumbar puncture. EDSS: Expanded Disability Status Scale.
